# Jaw size variation is associated with a novel craniofacial function for galanin receptor 2 in an adaptive radiation of pupfishes

**DOI:** 10.1098/rspb.2023.1686

**Published:** 2023-10-25

**Authors:** M. Fernanda Palominos, Vanessa Muhl, Emilie J. Richards, Craig T. Miller, Christopher H. Martin

**Affiliations:** ^1^ Department of Integrative Biology, University of California, 3101 Valley Life Sciences Building, Berkeley, CA 94720, USA; ^2^ Museum of Vertebrate Zoology, University of California, Berkeley, CA 94720, USA; ^3^ Department of Ecology, Evolution, and Behavior, University of Minnesota, Minneapolis, MN, USA; ^4^ Department of Molecular & Cell Biology, University of California, Berkeley, CA, USA

**Keywords:** gene expression, galanin, trophic morphology, craniofacial divergence, evo-devo, adaptive radiation

## Abstract

Understanding the genetic basis of novel adaptations in new species is a fundamental question in biology. Here we demonstrate a new role for galr2 in vertebrate craniofacial development using an adaptive radiation of trophic specialist pupfishes endemic to San Salvador Island, Bahamas. We confirmed the loss of a putative Sry transcription factor binding site upstream of galr2 in scale-eating pupfish and found significant spatial differences in galr2 expression among pupfish species in Meckel's cartilage using *in situ* hybridization chain reaction (HCR). We then experimentally demonstrated a novel role for Galr2 in craniofacial development by exposing embryos to Garl2-inhibiting drugs. Galr2-inhibition reduced Meckel's cartilage length and increased chondrocyte density in both trophic specialists but not in the generalist genetic background. We propose a mechanism for jaw elongation in scale-eaters based on the reduced expression of galr2 due to the loss of a putative Sry binding site. Fewer Galr2 receptors in the scale-eater Meckel's cartilage may result in their enlarged jaw lengths as adults by limiting opportunities for a circulating Galr2 agonist to bind to these receptors during development. Our findings illustrate the growing utility of linking candidate adaptive SNPs in non-model systems with highly divergent phenotypes to novel vertebrate gene functions.

## 1. Introduction

Craniofacial developmental anomalies are the most common source of birth defects in humans, present in 1 out of 700 births [1–3]. While Mendelian craniofacial defects are well characterized (e.g. Treacher Collins syndrome [4], Apert syndrome [5] and Crouzon syndrome [6,7]), the developmental genetics of complex craniofacial defects, such as micrognathia, are poorly understood [8–14]. With the continued lowering costs of genomic sequencing and functional genetic tools, it is increasingly feasible to develop new genetic models for understanding human development and disease.

Understanding the genetic bases of naturally occurring, highly divergent adaptive phenotypes in novel systems that parallel human clinical variation, such as ‘evolutionary mutant’ models [15–18], provides a powerful approach combining the tractable functional investigations possible in vertebrate model systems with genome-wide association scans of small-effect regulatory loci underlying natural craniofacial diversity. In particular, the most remarkable diversity of vertebrate craniofacial morphology is represented in teleost fishes, often associated with their diverse and sometimes highly specialized modes of feeding (e.g. [19–24]).

Emerging fish model systems include the rapidly evolving East African and Cameroon cichlid radiations, in which a small number of genetic changes underlie immense morphological disparity [25–32] and the repeated parallel speciation of stickleback ecomorphs in glacial lakes [33–36]. These systems provide excellent examples of leveraging naturally occurring and highly divergent craniofacial phenotypes as ‘evolutionary mutants' or ‘evolutionary forward genetics’ models to gain novel insights into the genetics of natural human craniofacial variation [17,37–42].

**Figure 1.**
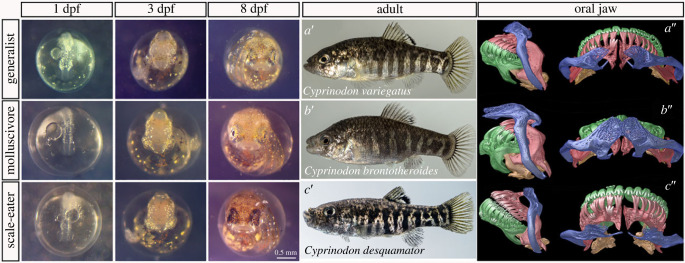
Divergent craniofacial morphology and development of the San Salvador Island *Cyprinodon* pupfish radiation. *C. variegatus* (first row) is a trophic generalist distributed across the western Atlantic and Caribbean; *C. brontotheroides* (second row) is a molluscivore and *C. desquamator* (third row) is a scale-eater, both endemic to the hypersaline lakes of San Salvador Island (SSI), Bahamas. Left panel: development at 1-, 3-, and 8-days post-fertilization (dpf). Middle panel: laboratory-reared adult female pupfishes of each species. Right panel: Lateral and dorsal views of µCT scans of craniofacial morphology (modified from [43]). The maxilla is coloured in blue, premaxilla in red, dentary in green, articular in orange.

Here we demonstrate the utility of an evolutionary radiation of *Cyprinodon* pupfishes for discovering and validating the craniofacial function of a new gene associated with jaw evolution via QTL and GWAS analyses. Pupfishes offer some advantages over other evolutionary fish systems because they (1) rapidly evolved highly divergent and unique craniofacial phenotypes (figure 1) with minimal genetic differentiation among species [43–50], (2) speciated in the face of ongoing gene flow resulting in very few highly differentiated genomic regions associated with species-specific craniofacial traits [51–57], and (3) are highly amenable to laboratory rearing and imaging due to their high fecundity, daily egg production, and egg transparency comparable to zebrafish [58,59]. This radiation contains the widespread algae-eating generalist pupfish, *Cyprinodon variegatus* (figure 1*a*), which is broadly distributed across the Caribbean and North American Atlantic coast and occurs in sympatry with two microendemic trophic specialist species found only in the hypersaline lakes of San Salvador Island (SSI), Bahamas. Each trophic specialist displays highly divergent behaviour, pigmentation and craniofacial morphology: the molluscivore *C. brontotheroides* has a novel nasal protrusion that is a skeletal extension of the maxilla and foreshortened robust oral jaws (figure 1*b*); and the scale-eater, *C. desquamator*, exhibits two-fold larger oral jaws and overall brachycephalic features (figure 1*c*) [46,47,60,61]. There is also a fourth intermediate scale-eating ecotype in some lakes [62].

Previous genomic and transcriptomic work on the SSI pupfishes identified dozens of new candidate craniofacial genes never previously characterized as craniofacial or directly investigated in other systems [54,56,57,62–67]. One of the most promising candidates was galanin receptor 2a, the second receptor type for the galanin peptide. Using a genome-wide association (GWA) test across 202 individuals from the SSI radiation and outgroup populations, we previously found an association of the regulatory region of *galr2a* with lower jaw length, confirming an earlier pilot study that found the *galr2a* region to be among the top five strongest associations with lower jaw length, containing highly differentiated SNPs between trophic specialist species [56,63]. An analysis of hard selective sweeps using both site frequency spectrum (SweeD) and linkage disequilibrium (Omegle) based summary statistics additionally found evidence of a putative adaptive allele in the 20 kb regulatory region upstream of *galr2* that swept to fixation in the scale-eater *C. desquamator* population on SSI 696–1008 (95% credible interval) years ago, potentially providing a pivotal stage in adaptation to scale-eating [56]. Furthermore, an independent quantitative trait loci (QTL) mapping study in F2 intercross hybrids between scale-eater and molluscivore parents found a significant QTL on linkage group 15 containing *galr2a* that accounted for 15.3% of the phenotypic variance in premaxilla length (*n* = 178) [67], with a positive effect on jaw length in the scale-eater genotype. Similarly, a second independent study of an F2 hybrid intercross from a second lake found evidence of a QTL in this region explaining 8% of the phenotypic variance in the length of the coronoid process on the articular bone of the lower jaw (jaw closing in-lever; *n* = 227; [57]). The combined strength of evidence for a role of *galr2a* in craniofacial development across independent analyses of GWA, QTL, selective sweeps and genetic differentiation between species indicated that this was one of our highest priority candidates for functional studies.

In humans, *galr2* is abundantly expressed within the hypothalamus and hippocampus of the central nervous system and in the heart, kidney, liver, colon and small intestine, and it has genetic associations with epilepsy and Alzheimer's [68,69]. Classified as an orexigenic (appetite stimulant) gene, *galr2* is expressed in the human hypothalamus and in the ventral telencephalon of larval and adult zebrafish [68,70,71]. Although a role of *galr2* in craniofacial development has not previously been reported in the literature, its ligand, the neuropeptide galanin (GAL), is highly expressed in bones from early to post-embryonic development [72,73], with demonstrated effects on bone mass [74], muscle contraction [75] and periodontal regeneration [76].

Here we used Sanger sequencing to confirm two highly differentiated SNPs between SSI specialist species detected in our previous genomic studies affecting two predicted transcription factor binding sites in the *galr2* regulatory region, characterized the divergent craniofacial expression of *galr2* across all three SSI pupfish species using *in situ* hybridization chain reaction (HCR) [77,78] at two key developmental timepoints, and demonstrated that treatment with two different Galr2 receptor inhibitors reduced Meckel's cartilage length and increased chondrocyte density dependent on the species' genetic backgrounds.

## 2. Results

### (a) Two highly differentiated SNPs are associated with *galr2a* transcription factor binding sites

**Figure 2.**
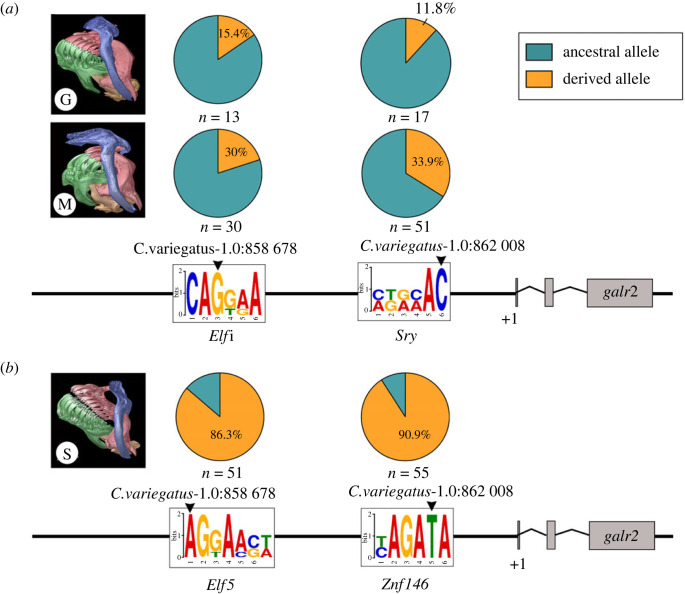
The two most differentiated single-nucleotide polymorphisms (SNP) between trophic specialists lie within the 20 kb regulatory region of *galr2a*. (*a*) Putative ancestral TFBS for *Elf1/Erg* and *Sry*, respectively, in the generalist (G), *Cyprinodon variegatus*, and the molluscivore (M) *Cyprinodon brontotheroides*. (*b*) Derived changes in predicted TFBS to *Elf5* and *Znf146* in the scale-eater (S) *Cyprinodon desquamator*. Pie charts indicate frequencies of ancestral (teal) and derived (orange) alleles at each locus. +1 represents the transcription starting site of *galr2a* (Galanin receptor 2a). µCT scans of craniofacial morphology are modified from [43]. Sequence logos of the position weight matrices (PWM) identified using MEME and TOMTOM.

Previous whole genome resequencing of over one hundred San Salvador Island (SSI) pupfishes identified only two highly differentiated single nucleotide polymorphisms (SNP) within the 20 kb regulatory region of *galr2a* between trophic specialists across different lake populations on SSI [56]. We designed primers (electronic supplementary material, table S5) and used Sanger sequencing to further genotype these SNPs in a large panel of wild-caught specialists from six lake populations. We confirmed the presence of a transversion from G to A approximately 11 kb upstream of the *galr2a* transcription starting site (TSS; figure 2*a*; electronic supplementary material, figure S1 and table S1), which changes a predicted CA**G**CAA *Elf1/Erg* transcription factor binding site (TFBS) to a predicted **A**GGGASW *Elf5* TFBS at this locus (using the Multiple Expectation maximizations for Motif Elicitation (MEME) server and the motif database scanning algorithm TOMTOM [79]). This transversion was observed in 86.3% of scale-eaters across four lake populations (*n* = 51) versus 20% of molluscivores across six lake populations on SSI (*n* = 30) (figure 2*a*, S1).

Using Sanger sequencing, we genotyped a second transversion from C to T approximately 8 kb upstream of *galr2a* TSS, which changes the predicted AGA**C**AA *Sry* TFBS to a predicted YAGA**T**A *Znf146* TFBS (figure 2*b*; electronic supplementary material, figure S2 and table S1). This transversion was observed in 90.9% of scale-eaters across six lake populations (*n* = 55) versus 33.9% of molluscivores across six lake populations on SSI (*n* = 53) (electronic supplementary material, figure S2). Notably, all scale-eaters sampled from Crescent Pond (CRP) contained the T transversion (*n* = 30) (electronic supplementary material, figure S2). The predicted TFBS changes in the *galr2a* cis-regulatory region across pupfishes, combined with a previous genetic mapping study that found a significant QTL in this region explaining 15% of phenotypic variance in oral jaw size [51], suggests that different spatial or temporal *galr2a* expression may underlie some of the craniofacial divergence in SSI pupfishes. Previous studies of allele-specific expression in SSI pupfishes were inconclusive due to lack of heterozygous sites in the *galr2a* transcripts [53].

### (b) Different spatio-temporal patterns of *galr2a* expression in craniofacial tissues

To determine if spatial or temporal changes in *galr2a* expression underlie SSI craniofacial divergence, we assayed *galr2a* expression in 2 dpf embryos and 8 dpf larvae from two independent lake populations for each of the three SSI species using fluorescent in-situ hybridization chain reaction (HCR) for *galr2a* and *tropomyosin 3b* (*tpm3b*), a component of thin filaments of myofibrils expressed in fish skeletal muscles [80], to visualize jaw and other cranial muscles. We also tested *galr2a* expression using orthogonal amplifiers labelled with three distinct fluorophores (see Methods for details) to ensure reliable detection of *galr2a* transcripts *in situ* across species and developmental stages (electronic supplementary material, video S1).

**Figure 3.**
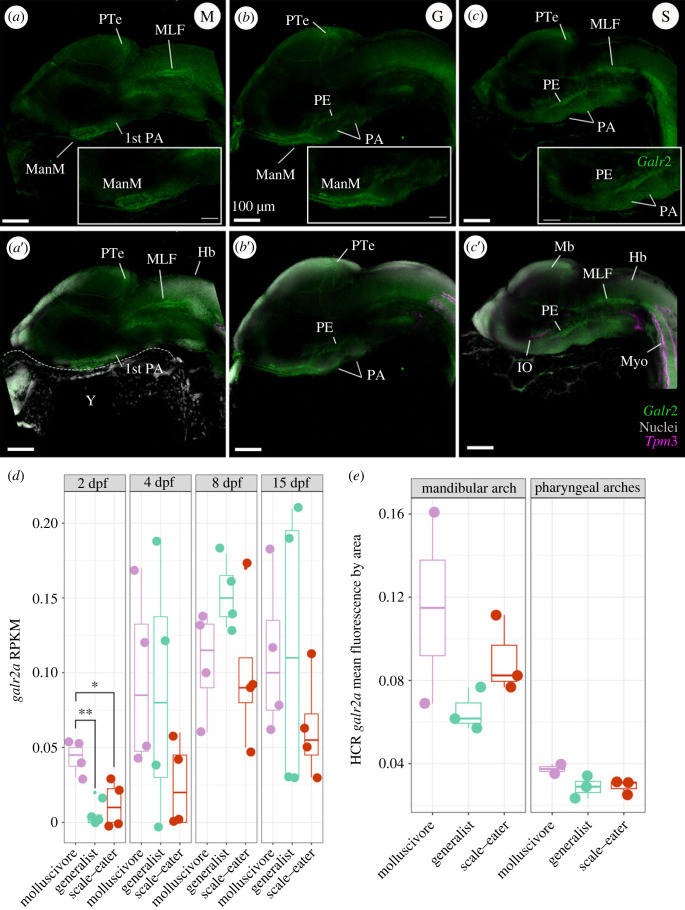
Spatio-temporal patterns of *galr2a* expression differ among SSI species at 2 dpf. Representative images of *Galr2a* (green) and *Tpm3b* (magenta) expression revealed by hybridization chain reaction (HCR) and DAPI nuclear staining (grey) in the: (*a*) molluscivore, *C. brontotheroides*; (*b*) generalist, *C. variegatus*; and (*c*) scale-eater, *C. desquamator*. (*d*) *Galr2a* reads per kilobase of transcript per million reads (RPKM) from an existing mRNAseq study sampling the entire head of each species at four developmental stages [81]. Galr2a showed significantly higher expression in the molluscivore relative to generalists (*p* = 0.003, Tukey's HSD test) and scale-eaters (*p* = 0.014, Tukey's HSD test) at 2 days post fertilization (dpf). (*e*) Mean fluorescence per area for *galr2a* quantified at 2 dpf in all three species showed similarly elevated levels of expression in the molluscivore for the mandibular and pharyngeal arches. Images shown are representative Z-stack maximum projection images of 30 optical sections taken every 3 µm from whole-mounted embryos imaged using a Zeiss LSM880 laser confocal microscope. PA: pharyngeal arches, PE: pharyngeal endoderm, ManM: mandibular mesenchyme, MLF: medial longitudinal fasciculus, PTe: posterior tectum, Di: diencephalon; Mb: midbrain, Hb: hindbrain, Y: yolk, Myo: somitic myofibers, IO: inferior oblique muscle.

At 2 dpf, *galr2a* expression was detected in broad regions of the central nervous system (e.g. the posterior tectum and medial longitudinal fasciculus) of specialists (figure 3*a,c*) and generalists, with apparent higher expression in both specialist species than in generalists. We also observed an apparent increase of *galr2a* expression anterior to the first pharyngeal arches in the molluscivore and generalist pupfishes relative to the scale-eaters.

**Figure 4.**
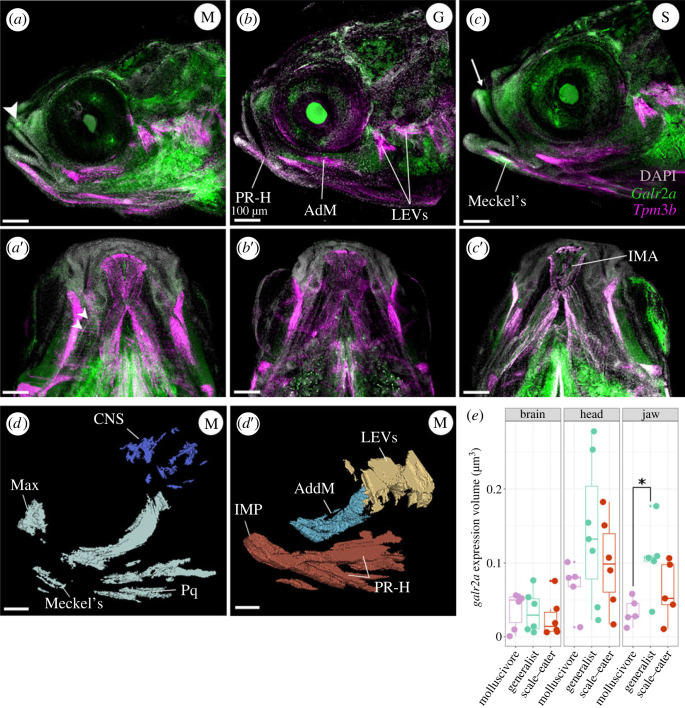
Spatio-temporal patterns of *galr2a* expression differ in molluscivores at 8 dpf. Representative images of *Galr2a* (green) and *Tpm3b* (magenta) expression revealed by hybridization chain reaction (HCR) and DAPI nuclear staining (grey). (*a*) Lateral view of an 8 dpf molluscivore (M in white circle), *(a*′) ventral view. (*b*) Lateral view of an 8 dpf generalist (G in white circle), (*b*′) ventral view. (*c*) Lateral view of an 8 dpf scale-eater (S in white circle), (*c*′) ventral view. The arrowhead in (*b*) and the arrow in (*c*) point to high expression of *galr2a* in the maxilla and premaxilla of the molluscivore and scale-eater specialists, respectively. White arrowheads in (*a*′) point to high expression of *galr2a* in the palatoquadrate of the molluscivore. *Galr2a* expression in the IMA was found only in the scale-eaters (*c*′). Images shown here are representative Z-stack maximum projection images of 30 optical sections taken every 15 µm from whole-mounted larvae imaged using a Zeiss LSM880 laser confocal microscope. (*d–d*′) 3D-Slicer view of the expression volume of *galr2a* (*d*) and *tpm3b* (*d*′) in an 8 dpf molluscivore pupfish larvae (*C. brontotheroides*). (*e*) Volume of *galr2a* expression in the brain (left panel), whole head (middle panel), and jaw (right panel) across SSI pupfishes. CNS: central nervous system, Max: maxilla. LEVs: levator arcus palatini and operculi, AdM (or AddM): adductor mandibular. IMA: intermandibularis anterior, PR-H: protractor hyoideus, Pq: palatoquadrate cartilage, Meckel's: Meckel's cartilage. Total volume rendered: 450 µm. All scale bars: 100 µm.

Using three-dimensional reconstruction and volume rendering analysis of HCR data for whole-mounted pupfishes at hatching time (8 dpf), we found that the *galr2a* expression domain was expanded in the jaws of the molluscivores relative to the generalists (figure 4; *p* = 0.03, Tukey's HSD), consistent with either greater tissue volume or an increased gene expression domain. By contrast, *galr2a* showed no differences in expression volume among species in the brain and head (figure 4; electronic supplementary material, table S3). G*alr2a* expression was detected in the Meckel's and palatoquadrate cartilages in all SSI pupfishes, in the premaxilla of the generalist and the scale-eater SSI specialist (figure 4*b–b*′,*c–c*′), the maxilla of the molluscivore SSI specialist (figure 4*a–a*′), and in the intermandibular muscles of the scale-eaters (figure 4*c*′).

**Figure 5.**
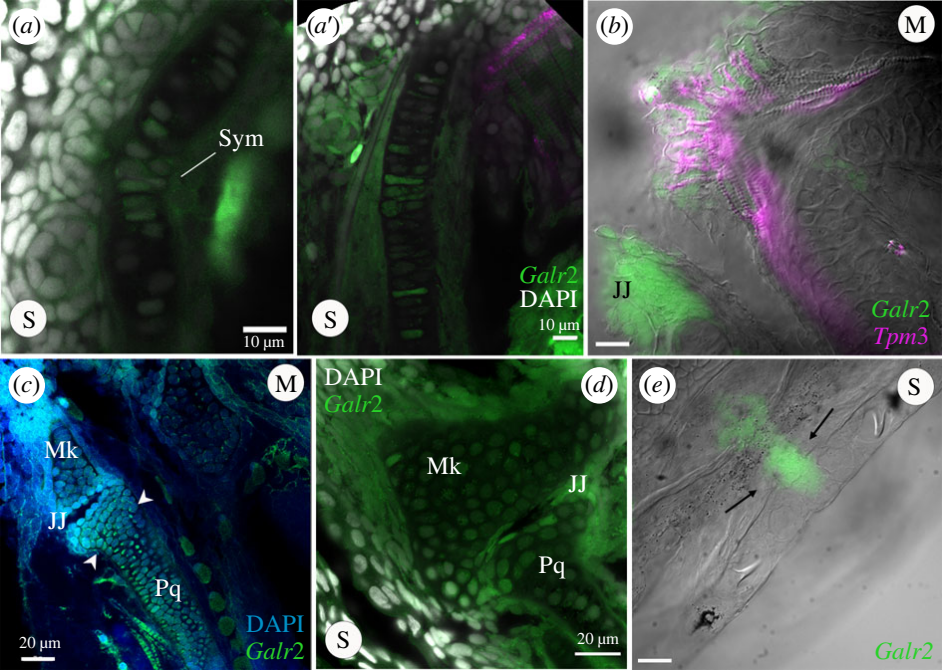
*Galr2a* is expressed in the chondrocytes of the Meckel's and palatoquadrate cartilages at hatching (8 dpf) in SSI pupfishes. HCRs showing tissue expression of *Galr2a* in green, and the muscle marker Tropomyosin 3b, (*Tpm3b*), in magenta. Nuclei are stained with DAPI (grey). (*a–a*′) Scale-eater *galr2a* expression in the chondrocytes of the Meckel's symphysis (Sym) and lateral side. (*b*) Ventral view of a molluscivore jaw at 8 dpf showing the expression of *Tpm3b* in the intermandibular muscles of the lower jaw. *Galr2a* is expressed in the jaw joint and the most anterior region of the lower jaw. (*c,d*) *Galr2a* is expressed in the chondrocytes of the Meckel's and palatoquadrate cartilages and around the jaw joint. (*e*) *Galr2a* expression in the premaxilla of a scale-eater. Unlabelled scale-bars: 10 µm.

At the subcellular level, *galr2a* mRNA was detected in the cytoplasm of chondrocytes at the Meckel's symphysis (figure 5*a*). By contrast, on the distal edge of the Meckel's cartilage expression was detected only in the cytoplasm of the elongated chondrocytes (figure 5*a*′). Towards the most posterior region of the Meckel's cartilage closest to the palatoquadrate cartilage, we observed *galr2a* expression in the cells surrounding the jaw joint (figure 5*b–d*; electronic supplementary material, video S1). We conclude that g*alr2a* was significantly differentially expressed in specific and distinct craniofacial tissues in the specialists at hatching time, suggesting an important role for craniofacial divergence in the SSI radiation.

We further tested for quantitative differences *in galr2a* expression at 2 dpf and 8 dpf by quantifying transcript counts from the only existing RNAseq data set for the heads of SSI pupfishes during development [81]. Only *galr2a*, but not *galr1a, galr1b, galr2a, galr2b* or *galanin*, showed significantly higher mRNA expression in the molluscivores relative to generalists (*p* = 0.003, Tukey's HSD test) and scale-eaters (*p* = 0.014, Tukey's HSD test) at 2 dpf (electronic supplementary material, table S2), whereas *galr2a* showed overall similar levels of expression in the head from 4 to 15 dpf in all three species (electronic supplementary material, table S2).

By using *Tpm3b* expression to label muscle cells, we observed at 2 dpf that *Tpm3b* expression was detected in somitic myofibers in all SSI pupfishes (figure 3*a*″–*c*″). However, only in the scale-eaters, *Tpm3b* expression was detected in the eye's inferior oblique muscle primordial cells (figure 3*c*″). At hatching time, *Tpm3b* expression was detected in all larval head muscles (figure 4*a*-*a*′, *b*-*b*′, *c*-*c*′; figure 5*b*). The scale-eater and molluscivore *Tpm3b* expression volume was significantly larger than in the generalists (electronic supplementary material, table S3; *p* < 0.05; 1-way ANOVA, Tukey's HSD test).

### (c) Chemical inhibition of Galr2 receptors affects Meckel's cartilage length and chondrocyte density

**Figure 6.**
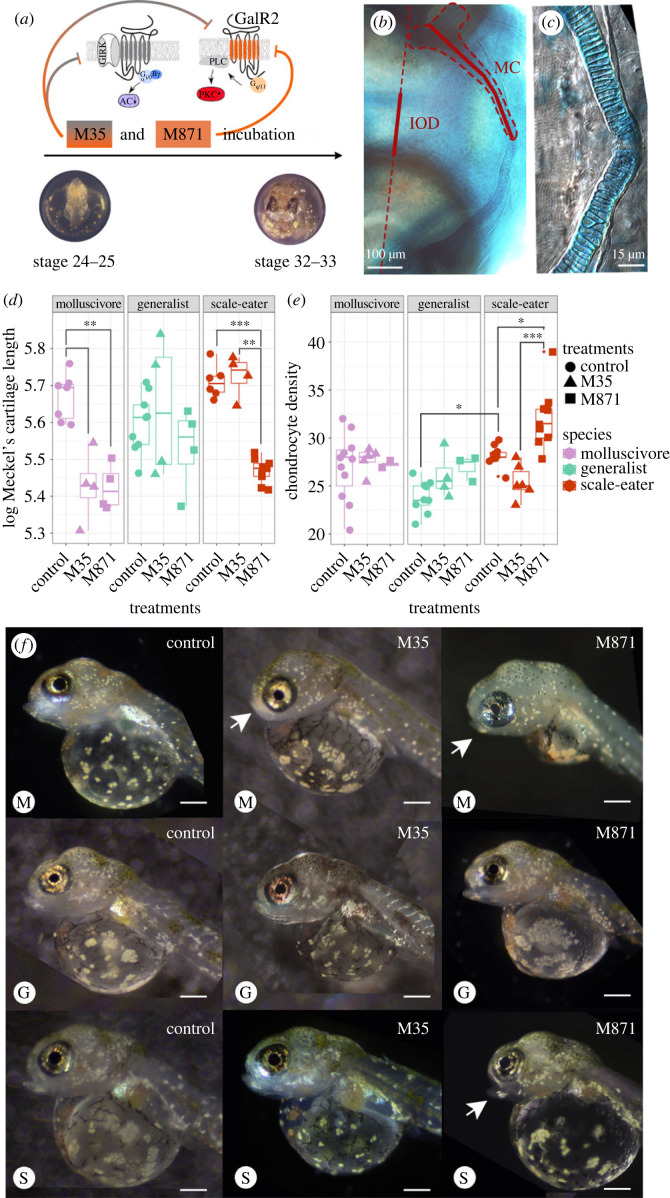
Galr2 receptor inhibition reduced Meckel's cartilage length in both specialists and increased chondrocyte density in scale-eaters. (*a*) Galr2 and Galr1 + 2 inhibition protocol during pupfish development. *C. desquamator* embryos are depicted. (*b*) Alcian blue cartilage staining at Stage 32–33. IOD: interocular distance, MC: Meckel's cartilage. The MC length and IOD are shown in red. (*c*) Chondrocytes at the symphysis. (*d*) Changes in the log-transformed Meckel's length and the log-transformed interocular distance at stage 32–33 across species and treatments. (*e*) Chondrocyte numbers at the symphysis across species and treatments. *p < 0.05, **p < 0.01, ***p < 0.001 Tukey's HSD test. f. Representative control and treated larvae of each species at stage 32–33. Arrows point to abnormal lower jaws. M: molluscivores, G: generalist, S: scale-eaters. Scale-bars = 0.4 mm. Galr1 and Galr2 cartoons were modified from [85].

To study the effect of Galr2 in craniofacial development, we inhibited the endogenous activation of all four known galanin receptors in teleost fishes (*Galr1a* and b, *Galr2a* and b [68,82]) using M35 (Innopep, Inc.), a synthetic peptide antagonist of Galr1+2 galanin receptors [83], and M871 (Abcam), a Galr2-specific synthetic peptide antagonist [84]. Embryos of all three species from two different lake populations were exposed from stages 24–25 (2 dpf, with the appearance of the first pharyngeal arches) until hatching at stages 32–33 (8 dpf; figure 6*a*).

At approximate hatching time (8 dpf), both trophic specialist species raised under laboratory common garden conditions exhibited increased Meckel's cartilage length (figure 6*d*), consistent with the longer jaws of adult scale-eaters and more robust jaws of the molluscivore relative to the more gracile jaws of the generalist [47,59]. We found that exposure to M871, the Galr2-specific antagonist, significantly reduced the length of the Meckel's cartilage in both specialists relative to the generalists (figure 6*d*; *p* = 7.89 × 10^−5^ for scale-eaters; *p* = 0.001 for molluscivores; 2-way ANOVA, Tukey's HSD test) while the interocular distance remained unchanged between control and treated larvae (electronic supplementary material, figure S4). By contrast, M35 (Galr1 and Galr2 antagonist) only significantly reduced Meckel's length in the molluscivore (figure 6*c*). Meckel's length of generalists was unaffected by exposure to M35 or M871 (figure 6*d*).

To understand the cellular effect of Galr1 and 2 antagonists on Meckel's length across species, we quantified the number of chondrocytes 100 µm from the symphysis and the mean width of ten chondrocytes nearest to the symphysis. We found no significant differences in the mean chondrocyte width among pupfish species but observed increased chondrocyte density in untreated scale-eaters relative to generalists (figure 6*e*; electronic supplementary material, table S4; *p* = 0.009, ANOVA, Tukey's HSD test). Moreover, only scale-eaters responded to M871, but not M35, by further increasing chondrocyte density relative to the control larvae (figure 6*e*; electronic supplementary material, table S4; *p* = 0.03, ANOVA, Tukey's HSD test; mean ± 1 s.e.; control = 28.22 ± 0.38; M871 = 32.06 ± 1.06). Despite having shorter jaws after treatment with M871, chondrocyte density was significantly increased (figure 6*e*; mean ± 1 s.e.; control = 28.22 ± 0.38; M871 = 32.06 ± 1.06).

## 3. Discussion

We used an evolutionary radiation of trophic specialist pupfishes, endemic to San Salvador Island in the Bahamas, to discover a novel function for *galr2a* in craniofacial divergence. Specifically, we confirmed that two transcription factor binding sites upstream of *galr2a* display highly divergent allele frequencies between trophic specialist species, visualized *galr2a* expression in craniofacial tissues in all three SSI pupfish species at two developmental stages, and demonstrated a phenotypic effect on Meckel's cartilage length and chondrocyte density using synthetic peptides to inhibit the activity of Galr2 and Galr1 + 2. Our findings demonstrate a crucial role for Galr2 in craniofacial divergence within this pupfish radiation. Our study also provides a roadmap in a non-model vertebrate system for rapidly identifying previously uncharacterized candidate genes important for adaptation to novel ecological niches (e.g. trophic specialization) that can be quickly validated through classic and state-of-the-art developmental biology tools. Overall, our research contributes to understanding the genetic basis of phenotypic evolution and adaptation in non-model organisms.

### (a) Putative loss of a transcription factor binding site for *galr2a* in scale-eating pupfish

One of the most common evolutionary changes associated with phenotypic changes among closely related species is the gain or loss of cis-regulatory elements [86,87]. More than 85% of scale-eaters carry two transversions in the regulatory region of *galr2a* (figure 2). Combined with our observations of reduced *galr2a* expression in the mandibular mesenchyme during early jaw development in this species (figure 3), we conclude that the putative loss of a predicted *Sry* transcription factor binding site in scale-eaters is the most likely explanation for changes in gene expression, rather than a gain of a new predicted TFBS for *Znf416* at this locus (figure 2). This is further supported by the critical role of the *Sry*-related HMG box (Sox) family of transcription factors (especially the SoxE group including *Sox8, Sox9* and *Sox10*) in craniofacial development as *Sry* transcription factors specify the behaviour, multipotency and survival of neural crest cells during vertebrate development [88–92]. Moreover, the maintenance of the *Elf* (E74-like ETS transcription factor) family TFBS upstream of the loss of the *Sry* TFB in scale-eaters suggests that *Elf* TF recruitment to the regulatory region of *galr2a* may play a broader role in head development than species-specific craniofacial differences. Interestingly, morphant zebrafish larvae for *elf3* show craniofacial cartilage defects [93], suggesting still unexplored possible roles for *elf* genes in craniofacial development and evolution.

Along with the identification of an *Sry* TFBS site using MEME, the Sry-related HMG box sox21 and sox7 were the second and third top-hits for predicted TFBS for the galr2a downstream SNP in molluscivores and generalists (TFBS predicted to be lost in scale-eaters). We checked both sox21 and sox7 expression through time and across species and found that they are mostly expressed at 2 and 4 dpf, with sox21b expressed more than sox7 (greater than 25 RPKM for sox21b, greater than 3 RPKM for sox7). We did not find species differences across time for sox21b; however, sox7 expression was significantly higher in scale-eaters than in molluscivores and generalists at 2 dpf (electronic supplementary material, table S2).

Alternatively, we cannot rule out a gain of a TFBS upstream of *galr2a* in scale-eaters, additive or epistatic effects of both upstream transversions, or more complex regulatory architectures, such as many interacting functional cis- and trans-acting regulatory variants or combinations of variants segregating at lower frequencies in trophic specialists that we have not prioritized [53,94,95]. However, our mapping cross of a single outbred pair of trophic specialists indicates that a single moderate-effect QTL containing *galr2a* explains 15% of phenotypic variation in oral jaw length between these species, consistent with causative variants affecting jaw size originating from this region [51].

### (b) Differential *Galr2a* expression during development is associated with craniofacial divergence in SSI pupfishes

We found *galr2a* expression in our *in situ* hybridization experiments to be consistent with previously published RNAseq data for craniofacial tissues in this radiation (figure 3) [53,64,96–98], with *galr2a* being differentially expressed only at 2 dpf. We observed distinctive spatial *galr2a* expression among SSI species at 2 and 8 dpf suggesting important time and tissue-specific regulation of *galr2a* expression during pupfish development. At 2 dpf, we found a strong association between decreased expression of *galr2a* in the mandibular mesenchyme anterior to the first pharyngeal arch with the future Meckel's cartilage and oral jaw lengths in the adults of each species; with increasing *galr2* abundance in the molluscivores associated with shorter, but more robust jaws in adults [47,59]. By contrast, the reduced *galr2a* expression in the mandibular mesenchyme is associated with the development of longer oral jaws in scale-eaters, which is apparent as early as hatching time (figure 6*d*) [44].

We noted expression of *galr2a* in the maxilla of only molluscivores at hatching (figure 4*a–c*), absent in the scale-eaters and generalists, consistent with the uniquely enlarged and anteriorly protruding head of the maxilla in this species [43,47,49,50,59,98]. Furthermore, *galr2a* expression in the intermandibular muscles of the scale-eaters at hatching time (figure 4*c*) suggests that *galr2a* expression can also modulate the development of the observed hypertrophic musculature of the adductor mandibulae in the adult scale-eating pupfish [43]. Altogether, these interspecific differences in spatial expression support a novel role for *galr2a* in musculoskeletal development and may contribute to the divergent craniofacial morphology observed in SSI pupfishes.

### (c) Receptor inhibition supports a novel function for GALR2 in craniofacial divergence of SSI pupfishes

Interestingly, the response of Meckel's cartilage length and chondrocyte density to the inhibition of Galr-receptor pathways was highly dependent on the species' genetic background. Scale-eaters responded only to the Galr2-specific antagonist M871 with substantially reduced Meckel's cartilage length and increased chondrocyte density but were unaffected by the Galr1 + 2 antagonist M35 (figure 6*c*). Importantly, M871 exhibits a higher binding constant for Galr2 receptors than the Galr1 + 2 antagonist M35 [84,99]. Previous transcriptomic data from craniofacial tissues during early development [96] also indicates that *galanin* and *Galr1* mRNA transcript abundance does not vary among the three SSI species (electronic supplementary material, table S3).

**Figure 7.**
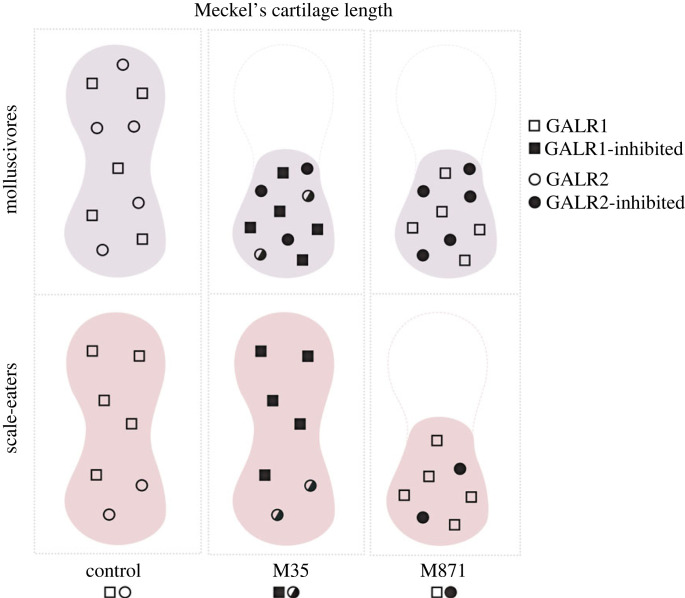
Proposed mechanism for how galanin receptor abundance affects Meckel's cartilage length in the two pupfish trophic specialists after exposure to M35 (weak Galr1 + Galr2 antagonist) or M871 (strong Galr2 antagonist). Molluscivores (top row) express more *galr2a* at 2 and 8 dpf than scale-eaters (bottom row) in their Meckel's cartilage (see figures 3 and 4), resulting in more Galr2 receptors (open circles). Increased Galr2 receptor putative abundance in molluscivores results in their reduced Meckel's cartilage length (figure 6) due to inhibition by both the weak antagonist (M35: middle column; half-filled circles) and strong antagonist (M871: right column; filled circles) of Galr2. By contrast, reduced Galr2 receptor putative abundance in scale-eaters (bottom row) results in their reduced Meckel's cartilage length only after exposure to the strong Galr2-specific antagonist M871.

Therefore, we conclude that even though scale-eaters expressed the lowest amount of *galr2a* transcripts and presumably contain overall fewer Galr2 receptors in their craniofacial tissue, M871's higher binding affinity [85] was sufficient to inhibit these fewer receptors, resulting in reduced Meckel's cartilage length (figure 7). By contrast, the presumably greater concentration of Galr2 receptors in molluscivore craniofacial tissue due to increased Galr2 expression during development resulted in the inhibition of these receptors by both M871 and M35, despite its lower binding affinity for Galr2 [85]. We also conclude that inhibition of Galr1 receptors by M35 does not affect Meckel's cartilage length or chondrocyte density and that the specific inhibition of Galr2 by M871 was sufficient to drive equal Meckel's cartilage length shortening. Finally, the generalists' lack of significant response to M35 is consistent with their decreased levels of *galr2a* at 2 dpf, suggesting that, as in scale-eaters treated with M35, fewer Galr2 receptors may be present for inhibition in these species’ background. However, the response to M871 appears to be specialist-specific, with little effect on the generalist Meckel's cartilage length, and thus, we cannot rule out more complex species-specific factors interacting with Galr regulatory pathways.

Our work is consistent with previous work showing that *Galr2* activation inhibits cell proliferation in neuronal cell lines [85,100], suggesting that the TFBS allele frequency shifts observed in scale-eaters are the cause for low *galr2a* abundance and, consequently, have low Galr2 activity during craniofacial development resulting in increased chondrocyte density and Meckel's cartilage length. Thus, the increased chondrocyte density observed in the M871-treated scale-eater larvae likely results from the strong and continued inhibition of the fewer Galr2 receptors available during development due to their genetic background. Lastly, due to the variable expression of *galr2a* between species at stage 24 but similar expression in chondrocytes of the Meckel's and palatoquadrate cartilages of different species at stage 33, we speculate that galr2a may have a species-specific morphogenetic role during pharyngeal arch differentiation and early jaw development and an osteogenic role at later stages.

In conclusion, we propose that the inhibition of Galr2 and not Galr1 induces the reduction of Meckel's cartilage length. Thus, a greater number of Galr2 receptors in the molluscivore specialist due to increased expression of *galr2a* during early development may result in their reduced oral jaw lengths as adults through increased opportunities for endogenous agonistic binding interactions (figure 7). Fewer Galr2 receptors on the scale-eater jaw may result in their enlarged jaw lengths as adults by limiting opportunities for Galr2 endogenous agonists to bind to these receptors during development. In the generalists, a greater number of Galr2 receptors but smaller overall Meckel's cartilage length may limit the sensitivity of this species to Galr2 and Galr1 + 2 endogenous agonists, however, resulting in their intermediate length and least robust oral jaws among the three species under control conditions [43,46].

## 4. Conclusion

Our results support a novel role of the second receptor for galanin, *Galr2*, as a craniofacial modulator gene important in controlling craniofacial development and interspecific divergence through modifying its transcript abundance and receptor activity. *Galr2a* transcript abundance changes among species are associated with genetic changes in the regulatory region of *galr2a*, consistent with the loss of a transcription factor binding site in the scale-eating pupfish. We propose a model in which reduced Galr2 receptor abundance in the oral jaws of scale-eaters results in fewer endogenous agonistic interactions, increasing Meckel's cartilage length presumably by decreased inhibition of chondrocyte proliferation (as a downstream effect of endogenous Galr2 activation). We also acknowledge the polygenic nature of jaw development and note that our previous genetic mapping experiments place an upper bound of 15% of oral jaw variation that may be due to differences in *galr2a* regulation among the myriad craniofacial genes driving the evolution of divergent craniofacial traits in this system.

## 5. Material and methods

Full details on pupfish husbandry, HCR FISH staining, microscopy, Galr2-inhibition, sequencing, TFBS prediction, gene expression and statistical analyses are available in the supplemental material.

## Data Availability

All data and R scripts used for this study are included as electronic supplementary material [101].
